# Fogs

**Published:** 1892-02-20

**Authors:** 


### PASSING TOPICS.

### FOGS.

LORD MIDLETON'S speech in the House of Lords upon the subject of fogs was remarkable chiefly for the impracticable nature of the remedies he propounded for a grave and universally acknowledged evil.

Of course, the noble Lord had little difficulty about making out a good case against the fog. Nobody can deny that such plague as Londoners have had to endure from time to time, during the last two years particularly, is worthy the attention of the legislature. Even as sources of mere inconvenience fogs have proved terribly irritating, and when we come to reckon up the full measure of their infliction—the loss of time, the loss of light, the loss of money, the loss of temper, the cessation of cab and omnibus traffic, the disorganization of railways, and the suffocation of weakly persons, we reach a total of evil directly attributable to them which is nothing short of appalling.

So far, all must be agreed, but when it comes to considering a remedy, our legislators do not give promise of much assistance. Lord Midleton did not even appear to remember that fogs of a kind are common enough all over our islands, and not at one season only. There is not a month of the year when, whether we travel N., E., S., or W., it is possible to feel quite safe from the invasion of the mists, which are only more tolerable outside big towns than in them, for the simple reason they are cleaner. It is by no means the case that the metropolis has a monopoly of fog. Only last mid-summer, the S. and E. coasts were enveloped for nearly 48 hours in a drifting sea fog accompanied by an icy wind, which sent the temperature down almost to freezing point. Yet London escaped, and whether or not, as the Prime Minister intimates, London fogs so-called have their origin in the Essex Marshes and the North Sea, it is certain they are not indigenous to the Metropolis, while it is equally certain that no matter how pure and innocent they may be outside the town, contact with London smoke and London exhalations speedily charges them with a poison and a pungency which to all but the most robust must be fraught with inconvenience and danger.

But unless we are able to remove the conditions of which fogs are bred, and not even the ambition of nineteenth century science is equal to attempting that, it is idle to talk or to write as if they were preventable evils. As reasonably talk of doing away with clouds, for what are fogs but earth-clouds ? or expect a man to be equally clear-headed, no matter what the exciting or depressing circumstances acting upon his brain, as to suppose that the exquisitely sensitive medium, the atmosphere, can be rendered independent of all the variations of temperature and other vicissitudes we comprehend in the term—weather.

If Lord Midleton sees his way to accept the Select Committee Lord Salisbury offered him upon the somewhat contemptuous condition that he should himself find members to serve upon it, good might follow, and any efforts in the direction of a remedy, if not too far-fetched and impracticable, would merit grateful appreciation by all classes, and especially by those who, as it has been pathetically observed, must remain to breathe the air of London whatever its composition.

It would be best, however, to start with the conviction that fogs we shall always have with us, and that what we have to do is to make the best of them. If they can be kept clear of deleterious matter, much as we might prefer their absence to their company, they will have purged themselves of their chief contempt for human comfort and well-being, and may be allowed to come and go with a pious resignation on our part, born of the reflection that, although nothing can make them altogether pleasant concomitants of society, they have at least ceased to be dangerous and disintegrating.

AGRICOLA.

